# Prevalence and determinants of allergic rhinitis among high school students exposed to industry allergens in Eastern Ethiopia

**DOI:** 10.1371/journal.pone.0324748

**Published:** 2025-06-02

**Authors:** Selamawit Gashaw, Ayantu Kebede, Gada Edea, Birhanu Yadecha, Moges Tesfaye, Hailu Merga

**Affiliations:** 1 College health Sciences, Arsi university, Asela, Ethiopia; 2 Department of Epidemiology, Institute of Health, Jimma University, Jimma, Ethiopia; 3 Department of Nursing, School of Health sciences Ambo University Waliso Campus, Waliso, Ethiopia; National Cheng Kung University, TAIWAN

## Abstract

**Introduction:**

Allergic rhinitis (AR), a common chronic disease triggered by allergens, often leads to school absenteeism in students. Investigating its magnitude and risk factors may play an important role in preventing the disease. Hence, this study aimed to assess the prevalence of allergic rhinitis and associated factors among high school students living in industrial areas in Eastern Ethiopia.

**Methods:**

A school-based cross-sectional study design was conducted in September 2023 among 442 randomly selected high school students. Data was collected using a structured self-administered questionnaire. Questions on allergic disease symptoms were based on the International Study of Asthma and Allergies in Children (ISAAC) core allergy and environmental questionnaire. Binary logistic regression analysis was done to identify the factors associated with allergic rhinitis. Finally, Adjusted Odds Ratio (AOR), 95% Confidence interval and P-value less than 0.05 was used to judge the statistical significance.

**Results:**

This study found that the magnitude of allergic rhinitis was 20.7% (95% CI: 17.0%, 24.8%). Similarly, this study identified that a family history of allergic rhinitis (AOR: 2.99, 95% CI: 1.42–6.29) and living with a smoker in the household (AOR: 3.63, 95% CI: 1.22–10.78) were significant risk factors. Conversely, living in a house far from traffic roads (AOR: 0.32, 95% CI: 0.15–0.67) and far from factories (AOR: 0.10, 95% CI: 0.03–0.31) were protective factors against allergic rhinitis.

**Conclusions:**

The current study found a lower magnitude of allergic rhinitis compared to the previous studies conducted in African countries. Having a smoker family member, distance between the house and nearest traffic road, distance between the house and nearest factory, and family history of allergy rhinitis were factors associated with allergy rhinitis. Hence, provision of health education to encourage people to stop smoking is strongly recommended. Similarly, persons with a family history of allergic rhinitis should minimize exposure to polluted environment and other trigger factors.

## Introduction

Allergic rhinitis (AR) is a global health problem that involves an immunoglobulin E (IgE)-mediated type 1 hypersensitivity response after exposure to certain allergens. Skin or in vitro testing that evaluates the presence of allergen-specific IgE (sIgE) can be used to determine sensitization to allergens. However, many sensitized individuals do not exhibit allergy symptoms, making it crucial to assess the association with clinical symptoms upon allergen exposure [1–3]. Medical history, physical examination, nasal endoscopy, and testing for allergen-specific IgE (skin prick tests or serum-specific IgE tests) are used to diagnose allergic reactions (AR) in certain patients [4].

Allergic rhinitis (AR) is one of the most common causes of rhinitis, affecting approximately 20% of the world population. Environmental factors in industrialized areas exacerbate allergic rhinitis by increasing inflammation, allergen exposure, and immune dysregulation. It has been demonstrated that children are disproportionately affected by the burden of Allergic rhinitis [2,5]. Uncontrolled AR may lead to serious illness and secondary diseases such as conjunctivitis, sinusitis, middle ear infections, jaw and teeth development disorders, and asthma. A study conducted at Medical College of Augusta University in USA revealed the negative impact of AR symptoms on daily activities, health status, emotional well-being, physical health, sleep performance, and academic achievement [6].

Evidence indicates that the magnitude of AR varies significantly between populations in industrialized and developing nations. The incidence rates have been shown to be increasing in high income countries and among low-to-middle income nations [7,8]. A study from Colombia found that the prevalence of self-reported allergic rhinitis was 30.8% among children and 36.6% among adolescents. Associated factors in children included current asthma and atopic dermatitis, use of acetaminophen and antibiotics in the first year of life, maternal education, smoking at home, and caesarean delivery [9]. Similarly, findings from Nigeria and Zambia showed that the prevalence of allergic rhinitis was 22.8% and 10.3% respectively which typically affects children and adolescents [10,11]. However, to the best of our knowledge, no recent studies have been conducted on school students in Ethiopia, and the 15.3% prevalence from the 2003 study is outdated [12].

Although allergic diseases are very widespread, their exact causes are still unknown. A more urban lifestyle is frequently cited as a significant risk factor in developed nations for the development of allergy problems. This demonstrates how incidence rates differ in urban and rural areas even within a single nation [8,13].

The increasing prevalence of various allergy rhinitis can be attributed to environmental conditions such as indoor and outdoor pollution, climate change, and global warming [14]. Specifically, the main factors that are increasing the risks of AR are having other allergic diseases, a family history of allergy, and residence and working environment [15]. Genetic factors and general characteristics of individuals (age, gender, family size, socioeconomic status and life style related factors) also play a role in rhinitis development [3,16,17].

However, the majority of published data on allergies and rhinitis come from developed nations, and there is a relative shortage of information in populations of Sub-Saharan Africa, particularly in Ethiopia. Therefore, this study aimed to assess the magnitude of allergic rhinitis and associated factors among high school students in an environment polluted by tannery factories in Mojo town of Eastern Oromia, Ethiopia.

## Materials and methods

### Study design and setting

This is a cross-sectional self-administered questionnaire study conducted in 18–30 September 2023 involving a total of 3883 grade 9–12 student population from two high schools (government (663) and private school (3220)) in Mojo town of Eastern Shewa Zone of Oromia region, Ethiopia. Mojo is an industry town located 57 km from Addis Ababa, the Ethiopian capital, with a population of 49,521. The temperature typically ranges from 56^o^F to 87^o^F and the town is situated at an elevation of 1,788 meters above sea level. Mojo town hosts several major manufacturing industries, including tanneries, textiles, edible oil factories, meat processing and packing plants, metal industries, and wood processing facilities, with tanneries being the most prominent.

### Population and sampling

The study population comprised of active students studying at the two Mojo High Schools during the study period. Students who were absent during the study period were excluded from the study. The sample size for this study was calculated using a single population proportion formula with the following assumptions: 95% confidence interval, 5% margin of error, and 50% proportion of allergic rhinitis since there was no previous study with a similar population. Hence, with the addition a 10% non-response rate, the final sample size became 442. Both high schools in Mojo Town were selected for this study. The final sample was selected proportionally from each school using a simple random sampling technique (lottery method) after obtaining a name list of all students.

### Study instrument and measurement

Allergic rhinitis (AR) was assessed in this study based on participants self-reported experience of typical symptoms, such as sneezing, a runny, blocked, or itchy nose, unrelated to upper respiratory infections within the last 12 months (presence or absence of these symptoms). Participants were classified as having AR if they had ever visited a health institution for symptoms related to allergic rhinitis or if they reported having been previously diagnosed with AR by a health professional (dichotomized as ‘yes’ and ‘No’ response) [18].

### Data collection and quality assurance

The instrument for data collection was developed after a review of relevant literature and questions on allergic disease symptoms were based on the International Study of Asthma and Allergies in Children (ISAAC) [1,3,8,13,18]. The questionnaire was prepared in English and translated to the participant`s languages, Afaan Oromo and Amharic, for clarity and testability. Then, it was back translated to English to check for consistency. The structured self-administered questionnaire was used, and the questionnaires consisted of socio-demographic and socioeconomic information, screening questionnaire of AR (symptoms, timing of symptom appearance, and history of diagnosis, frequency of symptom, disease severity and trigger factors of symptom), AR risk factors (environmental, socio-demographic, family history and lifestyle related factors) (S1).

Data were collected by two bachelor degree holder Nurses and supervised by public health experts who had experience on data collection and supervision. The data was collected at each school site before the day of the class ended through the self-administered questionnaire after explaining the objective of the study to the participants by data collectors. To ensure the quality of collected data, pretest was done on 5% of the total sample size of the study among Nafiad high school students in Bishoftu town, nearby study area. Questions with low response rate were identified, reviewed and modified accordingly. The data collectors and supervisor were trained for two days on the objectives of the study, its importance, how to collect the data, how to check data for its completeness and consistency and on confidentiality of information.

### Data analysis

Data were entered into Epi-Data version 3.1 and exported to SPSS version 26 for cleaning and analysis. Descriptive analyses like frequency, percentage distribution, and mean were conducted. The result was presented by the text and table. Bivariable logistic regression was done to select candidate variables for the final model with p-value less than 0.25 as a cut-off point. The Adjusted Odds Ratio was used as a measure of the strength of association, and a p-value of < 0.05 and 95% confidence interval were considered to declare statistical significance. Variance inflation factors (VIF), and the value of all independent variables was less than five, indicating the absence of strong multicollinearity. The general model fitness of the final model was checked with the Hosmer and Leme show goodness of fit tested, and a p-value > 0.6 indicates adequate model fitness.

### Ethical approval

An ethical approval was obtained from the Institutional Review Board (IRB) of Institute of Health, Jimma University (Ref. No. JUIH/IRB/480/23). Letter of support was also taken from Mojo Education office and communicated with those schools included in the study. Then, before the onset of the study, permission was sought from each study participant and oral informed consent/assent was obtained from study participants/parents/guardians/ in case of participants aged below 18 years. Participants were informed about the voluntary nature of participation and their right to discontinue at any moment if the questionnaire caused them any discomfort. Codes were used during data collection instead of personal details and identities.

## Results

### Sociodemographic characteristics of respondents

Of the 442 study sample identified, only 430 students participated in the study data collection making the response rate 97.3%. The mean (SD) age of the respondents was 17.36 (± 1.74) years. Most of them (61.6%) were between 17 and 19 years old, and more than half 254 (59.1%) of them were females. The majority 132 (30.7%) of the respondent`s family`s education status was able to read and write (Table 1).

**Table 1:** Socio-demographic characteristics of high school students in Mojo Town, Oromia, Ethiopia, 2023.

Variables	Frequency	Percentage
Child’ sex		
Male	176	41.6
Female	254	59.1
Age		
14-16 years	133	30.9
17-19 years	265	61.6
20 -24 years	32	7.4
Father’s occupation (n = 423)^*^		
Farmer	180	42.6
Merchant	73	17.3
Office work	49	11.6
Driver	27	6.4
Factory employee	26	6.1
Teacher	8	1.9
Unemployed	53	12.5
Others	7	1.7
Mother’s occupation (n = 418)^*^		
Merchant	91	21.8
Farmer	53	12.7
Office work	27	6.5
Factory employee	18	4.3
Teacher	5	1.2
House wife	20	4.8
Duration of living in current place (n = 430)		
Less than 5 years	95	22.1
5 years and more	335	77.9

*Note: The Ns for fathers and mothers do not sum to 430 because some students did not report having a mother and/or father, or did not provide information about them.

### Life style behaviour and environmental related factors

About eight percent 34 (7.9%) of those respondents had smoker family member and 3% of the respondents were also smokers themselves. Nearly two third 272 (63.3%) of them had pets in their house and more than two third 292 (67.9%) used insecticide to destroy insects. More than one-third 184 (42.8%) had cattle in their house. Eighty-seven percent of these students` family members used perfume and/deodorant. More than half of respondents (63.0%) used fuel mainly for cooking (Table 2).

**Table 2:** Life style behavior and environmental related factors among high school students in Mojo Town, Oromia, Ethiopia, 2023.

Variables	Frequency	Percentage
Household member who is smoker		
No	396	92.1
Yes	34	7.9
Do you smoke		
No	417	97.0
Yes	13	3.0
Have pets in the house		
No	158	36.7
Yes	272	63.3
Have cattle in the house		
No	246	57.2
Yes	184	42.8
Family member uses perfumes/deodorants		
No	55	12.8
Yes	375	87.2
Main source of energy for cooking		
Electricity	271	63.0
Charcoal	138	32.1
Gas cylinder	9	2.1
Kerosene	12	2.7
Use insecticide		
No	138	32.1
Yes	292	67.9
Distance between house and nearest traffic road		
100m and less	136	31.8
101-250m	67	15.7
251-500m	87	20.3
501-1000 m	93	21.7
More than 1000 m	45	10.5
Waste disposal		
Burn around the compound	117	27.2
Collected by municipality	111	25.8
In ditches and roads	99	23.0
Community dumps	62	14.4
Open fields	59	13.7
Others	19	4.4
Factory around their residence		
No	98	22.8
Yes	332	77.2
House distance from nearest factory		
< 1 km	38	11.4
1-3 km	73	22.0
3-5 km	74	22.3
≥ 5 km	147	44.3
Ever had food allergy		
No	370	86.0
Yes	60	14.0

### Prevalence of allergic rhinitis and comorbidity with other allergic diseases

In this study, the prevalence of allergic rhinitis was 20.7% (95% CI: 17.0%, 24.8%). Respondents who had ever experienced nasal symptoms without having a cold or flu were 124 (28.8%), and of these, 94 (75.8%) had nasal symptoms without having a cold or flu within the 12 months preceding the study. Nasal symptoms were accompanied by itchy and watery eyes in 49 (52.1%) of the cases. Most respondents experienced sneezing (63.8%), a watery and runny nose (50.0%), and nasal obstructions (44.7%). Dusty environments worsen symptoms in 69 (73.4%) of the cases. In most of the cases, nasal symptoms worsen at night (42.6%) and in the summer season (34.0%). In half of the cases (51.1%), nasal symptoms interfered with their daily activities within the 12 months preceding the study. Food allergies, asthma, and atopic dermatitis or eczema were found in 60 (14.0%), 33 (7.7%), and 105 (24.4%) of the students, respectively. Similarly, asthma, allergic rhinitis, atopic dermatitis, and food allergies were identified in 128 (29.8%), 98 (22.8%), 91 (21.2%), and 87 (20.2%) of the student`s family members, respectively (Table 3).

**Table 3:** Sign, symptoms and triggering factors of allergic rhinitis among high school students in Mojo Town, Oromia, Ethiopia, 2023.

Variables	Frequency	Percentage
Ever had problem with sneezing, runny or blocked nose without cold or flu		
No	306	71.2
Yes	124	28.8
In the past 12 months, had problem with sneezing, runny or blocked nose without cold or flu (n = 124)		
No	30	24.2
Yes	94	75.8
Nasal symptoms accompanied by itchy-watery eyes (n = 94)		
No	45	47.9
Yes	49	52.1
Nasal symptom worsens in dusty environment		
No	25	26.6
Yes	69	73.4
Time of nasal symptoms worsen		
Night	40	42.6
Day time	28	29.8
Early morning	27	28.7
All day	10	10.6
In the past 12-month, nasal problem interfered with daily activities (n = 94)		
No	46	48.9
Yes	48	51.1
Had food allergy (in the past 12months)		
No	370	86.0
Yes	60	14.0
Ever had dry, itchy skin with red rashes (eczema)		
No	325	75.6
Yes	105	24.4
Family member or close relative with asthma		
No	302	70.2
Yes	128	29.8
Family member or close relative with allergic rhinitis		
No	332	77.2
Yes	98	22.8
Family member or close relative with eczema		
No	339	78.8
Yes	91	21.2
Family or close relative with food allergy		
No	343	79.8
Yes	87	20.2

### Factors associated with allergic rhinitis

On multivariable logistic regression, having a smoker family member, distance between house and nearest traffic road, distance between house and nearest factory, and family history of allergic rhinitis were found to have statistically significant associations with allergic rhinitis among students (Table 4).

**Table 4:** Factors associated with allergic rhinitis among high school students in Mojo town, Oromia, Ethiopia, 2023.

Variables	Allergic rhinitis		COR (95% CI)	p-value	AOR (95% CI)	p-value
	Yes	No				
Ever been in nursery						
No	18 (9.5%)	172 (90.5%)	1.00		1.00	
Yes	71 (29.6%)	169 (70.4%)	4.01 (2.30,7.02)	.000	1.97 (0.82,4.73)	.129
Household member who is smoker						
No	69(17.4%)	327(82.6%)	1.00		1.00	
Yes	20(58.8%)	14(41.2%)	6.77 (3.26,14.06)	.000	3.63 (1.22,10.78)	.020^*^
Participate in physical or sport activities						
No	43(26.4%)	120(73.6%)	1.00		1.00	
Yes	46(17.2%)	221(82.8%)	0.58 (0.36,0.93)	.024	0.60 (0.29,1.27)	.182
Distance between house and nearest traffic road						
< 150 m	65 (38.7%)	103 (61.3%)	1.00		1.00	
>= 150 m	22 (9.5%)	210 (90.5%)	0.17 (0.10,0.28)	.000	0.32 (0.15,0.67)	.003^*^
Distance between house and nearest factory						
<1 1 km	27(71.1%)	11(28.9%)	1.00		1.00	
1-3 km	47(50.7%)	36(49.3%)	0.42 (0.18,0.97)	.042	0.53 (0.19,1.48)	.228
3-5 km	8(10.8%)	66(89.2%)	0.05 (0.02,0.14)	.000	0.10 (0.03,0.31)	.000^*^
>=5 km	7(4.8%)	140(95.2%)	0.02 (0.01,0.06)	.000	0.03 (0.01,0.10)	.000^*^
Family history of allergic rhinitis						
No	49(15.1%)	276(84.9%)	1.00		1.00	
Yes	40(38.1%)	65(61.9%)	7.83 (4.66,13.14)	.000	2.99 (1.42,6.29)	.004^*^
Family history of asthma						
No	48(15.9%)	254(84.1%)	1.00		1.00	
Yes	41(32.0%)	87(68.0%)	3.86 (2.30,6.48)	.000	0.72 (0.31,1.67)	.446
Personal history of asthma						
No	75(18.9%)	322(81.1%)	1.00		1.00	
Yes	14(42.4%)	19(57.6%)	2.39 (1.09, 5.24)	.030	2.53 (0.71,9.02)	.152

*Shows statistically significant at p-value <0.05 COR: Crude odds ratio, AOR: Adjusted odds ratio, CI: Confidence interval.

Students who were living with smoker household members were 3.6 times more likely to have allergic rhinitis than students without smoker household members (AOR: 3.63, 95% CI: 1.22, 10.78). Students who were living in a house that was more than 150 m away from the nearest traffic road experienced a reduction of 68% in the odds of having allergic rhinitis compared to students who were living within 150 m of the nearest traffic road (AOR: 0.32, 95% CI: 0.15–0.67). The odds of developing allergic rhinitis were 90% (AOR: 0.10, 95% CI: 0.03, 0.31) and 97% (AOR: 0.03, 95% CI: 0.01, 0.10) lower for those students who were living 3–5 km and more than 5 km away from the nearest factory, respectively, compared to students who were living with less than 1 km away from the nearest factory. Students with a family history of allergic rhinitis were about 3 times more likely to have allergic rhinitis than students without a family history of allergic rhinitis (AOR: 2.99, 95% CI: 1.42, 6.2).

## Discussion

This study assessed the prevalence of allergic rhinitis and its determinants in Eastern Oromia region of Ethiopia. Accordingly, the prevalence of AR was found 20.7% which matched with the report of allergic rhinitis of the global population (20%) [19], finding from Nigeria (22.8%) [10] and South Korea (25.5%) [20]. However, the result was lower than previous studies from Korea, Sudi Arabia, Egypt, and Senegal where the prevalence of allergic rhinitis was 30.2%, 47.7%, and 43% respectively. The difference may be due to industrial factors in Mojo town being less than those countries with large cities [18,21,22]. On the other hand, the prevalence of allergic rhinitis in this study was higher than the findings from Tanzania (10.3%), Yemen (12.2%), and China (6.6%) [11,23,24]. This variation might be due to the different environmental factors in triggering allergen rhinitis.

Regarding factors associated with allergenic rhinitis, students who were living with smoker household members were 3.6 times more likely to have allergic rhinitis compared to students without smoker household members. This is similar with the finding from Turkey [25]. In this study, the students with a family history of allergic rhinitis were about 3 times more likely to develop allergic rhinitis than students without a family history of allergic rhinitis. These results were similar with a study found in Sudi Arabia that showed a family history of asthma were 3 times increased the likelihood of allergic rhinitis and respondents whose fathers were smoking were around 1.7 times more likely to develop allergic rhinitis [21]. This study was also similar to the finding from South Korea that revealed family history of allergic disease increases the risk of developing allergic rhinitis (OR 6.7; 95% CI 3.50–12.82) [20].

In this study, students who were living in houses that were more than 150 m away from the nearest traffic road reduced their risk of developing allergic rhinitis by 68% compared to a student who were living within 150 m of the nearest traffic road. The current study matched with a study found in southern Sweden that showed participants who were living within 100 m from a road traffic were more likely to develop allergic rhinitis [26]. In our study, the odds of developing allergic rhinitis decreased by 90% and 97% among students who were living within 3–5 km and more than 5 km away from different factories, respectively, compared to those students who were living in less than 1 km distance from the factory. This result matched the study found in Senegal [25].

Though it is the first study done in Ethiopia in similar setting, it has the following limitations. First, it was self-report which might be subjected to inaccuracies due to recall bias or misreporting. Second, the study did not support with laboratory tests of serum IgE test. Third, the study included previous history of child hood, family and relative related questions which may prone participants to recall bias and affect research findings. Fourth, the cross-sectional design limits the study’s ability to establish causal relationships, as exposure and outcome data were collected simultaneously, making it unclear whether the exposure preceded the outcome.

## Conclusion

The current study found a lower prevalence of allergic rhinitis compared to the previous studies conducted in Africa. The identified determinants in this study were family history of allergy rhinitis, having smoker household member, the distance between house and the nearest traffic road, and the distance between house and the nearest factory. Hence, encouraging and awareness creation for parents to stop smoking at home is highly recommended. Moreover, a person with a family history of allergic rhinitis and high susceptibility should avoid living near roads and factories. The town municipality and affluent industries should plant industries more than 2km distant from people residence areas and public institutions. The industries themselves should establish industrial liquid and solid waste disposal systems.

## Supporting information

S1 File Questionnaire English version. (DOCX)
