# Supplementary material for: Prevalence and determinants of allergic rhinitis among high school students exposed to industry allergens in Eastern Ethiopia
Source: PLoS One. 2025 Jun 2;20(5):e0324748. doi: 10.1371/journal.pone.0324748 (PMC12129331; doi:10.1371/journal.pone.0324748)
Supplement: S1 File — (DOCX) [file pone.0324748.s001.docx]

**Questionnaire: English Version**

**Part 1: Socio demographic characteristics of the participants.**

**In circle the number to answer the questions.**

| Code | Questions | Alternatives |
| --- | --- | --- |
| 101 | What is your school? | 1.School of excellence (Private)  2. Modjo high school (Gov’tal) |
| 102 | Sex | 1. Male  2. Female |
| 103 | Height (in centimeters or in meter) | _______________C.M/M |
| 104 | What is your religion? | 1. Muslim  2. Orthodox  3. Protestant  4. Other specify_______ |
| 105 | Number of family member including any person who lives with your family in your house | _______________________ |
| 106 | Educational status of your father | 1.illitrate  2. read and write  3. primary education  4. secondary education  5. diploma, degree or MA |
| 107 | Educational status of your mother | 1.illitrate  2. read and write  3. primary education  4. secondary education  5. diploma, degree or MA |
| 108 | What is your father’s occupation? | 1.Farmer  2. Fabrica employee  3. Office work  4. Merchant  5. Unemployed  6. other specify________ |
| 109 | What is your mother’s occupation? | 1. Farmer  2. Fabrica employee  3. Office work  4. Merchant  5. House wife  6. other specify________ |
| 110 | Monthly family income | _________________ birr |
| 111 | Where do you live now? (current place of living) | 1. Modjo or around modjo  2. Other Specify |
| 112 | How many completed years did you live in the above mentioned area? | ________________years |
| 113 | Have you ever been to a nursery? | 1. Yes  2. No |
| IF YOU HAVE ANSWERED “NO” PLEASE SKIP TO PART 2 QUESTIONS | | |
| 115 | If yes for how many years you ever been to a nursery? | _________________years |

**Part 2: Life style related questions**

| Code | Questions | | Alternatives |
| --- | --- | --- | --- |
| 201 | Is there a family member or any person living in your house who smoke cigarette? | | 1. Yes  2. No |
| 202 | Do you smoke cigarette? | | 1. Yes  2. No |
| 203 | Do you engage in physical or sport activities such as football, hand ball, running etc? | | 1. Yes  2. No |
| **IF YOU HAVE ANSWERED “NO” PLEASE SKIP TO QUESTION 205** | | | |
| 204 | How many days per a week do you engage in physical or sport activities such as football, hand ball, running….? | | __________________days |
| 205 | Do you have difficulty of breathing when you engage in physical or sport activities such as football, hand ball, running….? | | 1. Yes  2. No |
| 206 | Do you have pets (cats or dogs or dove) in your house? | | 1.Yes  2. No |
| 207 | Do you have cattles (sheep, goat or cows…) in your house? | | 1. Yes  2. No |
| 208 | Do you watch Television? | | 1. Yes  2. No |
| 209 | If your answer is yes, averagely how many hours do you spend with watching Television per day? | ___________________hours | |
| 210 | Do you have smart phone? | 1. Yes  2. No | |
| 211 | If yes averagely how many hours do you spend with using other social media (Facebook, Telegram, Instagram…) per day? | ____________________hours | |
| 212 | Do you share bed with your siblings? | 1. Yes  2. No | |
| 213 | Do you use perfume, deodorant or soap? | 1. Yes  2. No | |
| 214 | If yes, how often do you use perfume, deodorant or soap? | 1. Always  2. Sometimes  3. Most of time | |
| 215 | Is there a family member who uses perfume, deodorant? | 1. Yes  2. No | |
| 216 | If yes, how often does your family member use perfume, deodorant? | 1. Always  2. Sometimes  3. Most of time | |
| 217 | What type of shoes you usually wear? | 1. Closed shoes  2. Sandals | |

**Part 3: Environmental related questions.**

| Code | Questions | Alternatives |
| --- | --- | --- |
| 301 | Do you live in your own house or rented house? | 1. Own house  2. Rented house |
| IF YOU HAVE ANSWERED “RENTED HOUSE” PLEASE SKIP TO QUESTION 303 | | |
| 302 | If you are living in your own house, How many completed years passed after the house is built? | ___________________years |
| 303 | If you are living in rented house, For how many completed years did you live the house you are currently living? | _____________________Years |
| 304 | How many windows does your house have? | _________________windows |
| 305 | How many rooms does your house have? | ________________rooms |
| 306 | Is there a carpet in your house of flour at any room? | 1. Yes  2. No |
| 307 | In your house what source of fuel is usually used for cooking? | 1.Electricity (Stove)  2. Charcoal  3. Kerosene  4. Gas cylinder  5. Other specify____________ |
| 308 | Do you or your family use insecticide to destroy insects like mosquito, cockroach, mites….? | 1. Yes  2. No |
| 309 | From what type of material did your house wall build? | 1. Clay mud and wood  2. Concrete Blocks  3. Brick  4. Other specify___________ |
| 310 | Is there a public toilet around your house? | 1. Yes  2. No |
| 311 | Where is your house location (Address)? | _______________ |
| 312 | How far the nearest traffic road from your house in estimated meters or kilo meters? (1 kilometer takes 13 to 15 minutes) | __________________M/kms |
| 313 | How often do trucks pass through the street where you live, on week days? | 1. Never  2. Seldom  3. Frequently through the day  4. Almost the whole day |
| 314 | In your community how the waste substances are disposed?  **(Multiple answers are possible)** | 1. In ditches and on roads  2. Open field  3. Burn around the compound  4. Community dump  5. Collected by municipality  6. Other specify___________ |
| 315 | How long does it take from your house to Modjo river on foot?  (1 kilometer takes 13 to 15 minutes) | _____________Minutes/Hour |
| 316 | Is there any factory in the surrounding that you are living now? | 1. Yes  2. No |
| 317 | If your answer is “yes” how far is the factory from your house in estimated meters or kilo meters?  (1 kilometer takes 13 to 15 minutes) | ____________________M/kms |

**Part 4: Screening questions**

| 401 | Have you ever had a problem with sneezing, or a runny, or blocked nose when you DID NOT have a cold or the flu? | 1. Yes  2. No |
| --- | --- | --- |
| **IF YOU HAVE ANSWERED “NO” PLEASE SKIP TO QUESTION 409** | | |
| 402 | In the past 12 months, have you had a problem with sneezing, or a runny, or blocked nose when you DID NOT have a cold Or the flu? | 1. Yes  2. No |
| **IF YOU HAVE ANSWERED “NO” PLEASE SKIP TO QUESTION 409** | | |
| 403 | In the past 12 months, has this nose problem been accompanied by itchy-watery eyes? | 1. Yes  2. No |
| 404 | Which symptom do you experience?  **(Multiple answers are possible)** | 1. Watery runny nose  2. Sneezing (violent/in bouts)  3. Nasal obstruction (inability to breath),  4. Itchy nose with watery/red itchy eyes |
| 405 | How many times per week do you experience those symptoms? | 1. less than 4 times per week  2. greater than 4 times per week |
| 406 | Do you feel that your nasal symptoms worsen in dusty environment? | 1. Yes  2. No |
| 407 | In which of the past 12 months did this nose problem worsen?  **(Multiple answers are possible)** | 1 Spring (Sept, Oct., Nov)  2. Winter (Dec, Jan, Feb)  3. Autumn (Mar, Apr, May)  4. Summer (June, July, Aug)  5. All through the year |
| 408 | In the past 12 months, did this nose problem interfere with your daily activities such as sleeping sports, leisure, and school performance? | 1. Yes  2. No |
| 409 | Have you ever had health professional (physician) diagnosed Allergy rhinitis? | 1. Yes  2. No |
| 410 | Do you have food allergy? (Swelling of lips, face, tongue and throat, skin rash, shortness of breathing, tingling or iching mouth and difficulty of breathing…) after the ingestion of certain foods. | 1. Yes  2. NO |
| 411 | Do you have health professional (physician) diagnosed Asthma? | 1. Yes  2. NO |
| 412 | Have you ever had dry, itchy skin with red rashes and bumps on your skin? | 1. Yes  2. NO |
| 413 | In the past 12 month, How many times did you infected with a common cold or flu? | ___________________times |
| 414 | Is there a family member or any close relative (aunt, uncle or grandparents in both sides) who had Asthma? | 1. Yes  2. NO |
| 415 | Is there a family member or any close relative (aunt, uncle or grandparents in both sides) who had Allergy rhinitis? | 1. Yes  2. NO |
| 416 | Is there a family member or any close relative (aunt, uncle or grandparents in both sides) who had dry, itchy skin with swelling, red rashes and bumps on skin? | 1. Yes  2. NO |
| 417 | Is there a family member or any close relative (aunt, uncle or grandparents in both sides) who had Food allergy? | 1. Yes  2. NO |
